# Insight on the evaporation dynamics in reducing the COVID-19 infection triggered by respiratory droplets

**DOI:** 10.1063/5.0057045

**Published:** 2021-07-07

**Authors:** Sumit Kumar

**Affiliations:** Advanced Technology Development Centre, Indian Institute of Technology Kharagpur, Kharagpur, West Bengal 721302, India

## Abstract

In this paper, the lifetime of coronavirus infected droplets under a stick-slip evaporation mode has been investigated, which may play a pivotal role in reducing the spread of COVID-19 infection. It is showed that the survival time of the virus can be reduced by increasing the receding contact angle or by reducing the initial contact angle of a drop deposited on a solid surface. It has been found that the lifetime of the virus increases almost five times under highly humid conditions as compared to dry conditions. It is further observed that the normalized lifetime does not depend upon thermo-physical properties, ambient temperature, relative humidity, and initial drop volume. A model has been proposed to estimate the shear stress acting on a virus taking into account the effect of a Marangoni flow. The presented model unveils that the magnitude of computed shear stress is not enough to obliterate the virus. The findings of the present model have been discussed in the context of reducing the COVID-19 infection, but the model can also be applied for coughed/sneezed droplets of other infectious diseases. Moreover, this physical understanding of evaporation dynamics on solid surfaces with a stick-slip mode may help in better design of a face mask, PPE kit, and other protective equipment used in public places in order to minimize the chances of infection and tackle the current pandemic. However, the reported model for estimating the survival time of the virus does not consider the effect of the thermo-capillary convection (the Marangoni effect).

## I. INTRODUCTION

The current pandemic COVID-19, known as the twenty-first century's most pandemic disease, caused by coronavirus is shown to be transmitted among human beings through small micro-droplets and airborne means, and it has already infected and taken the lives of millions of people throughout the world.^1–4^ This COVID-19 has not only killed hundreds of thousands of people but also has adversely damaged the economic progress throughout the world and humanity as a whole.^5^ A significant number of available reports on COVID-19 has been devoted to understanding the spreading and alleviating the mechanism of the COVID-19 infection.^6–13^ Out of all the existing mechanisms of COVID-19 transmission, coughed or sneezed droplets resting on a solid surface play a very crucial role in spreading the virus infection.^1–4^

**FIG. 1.**
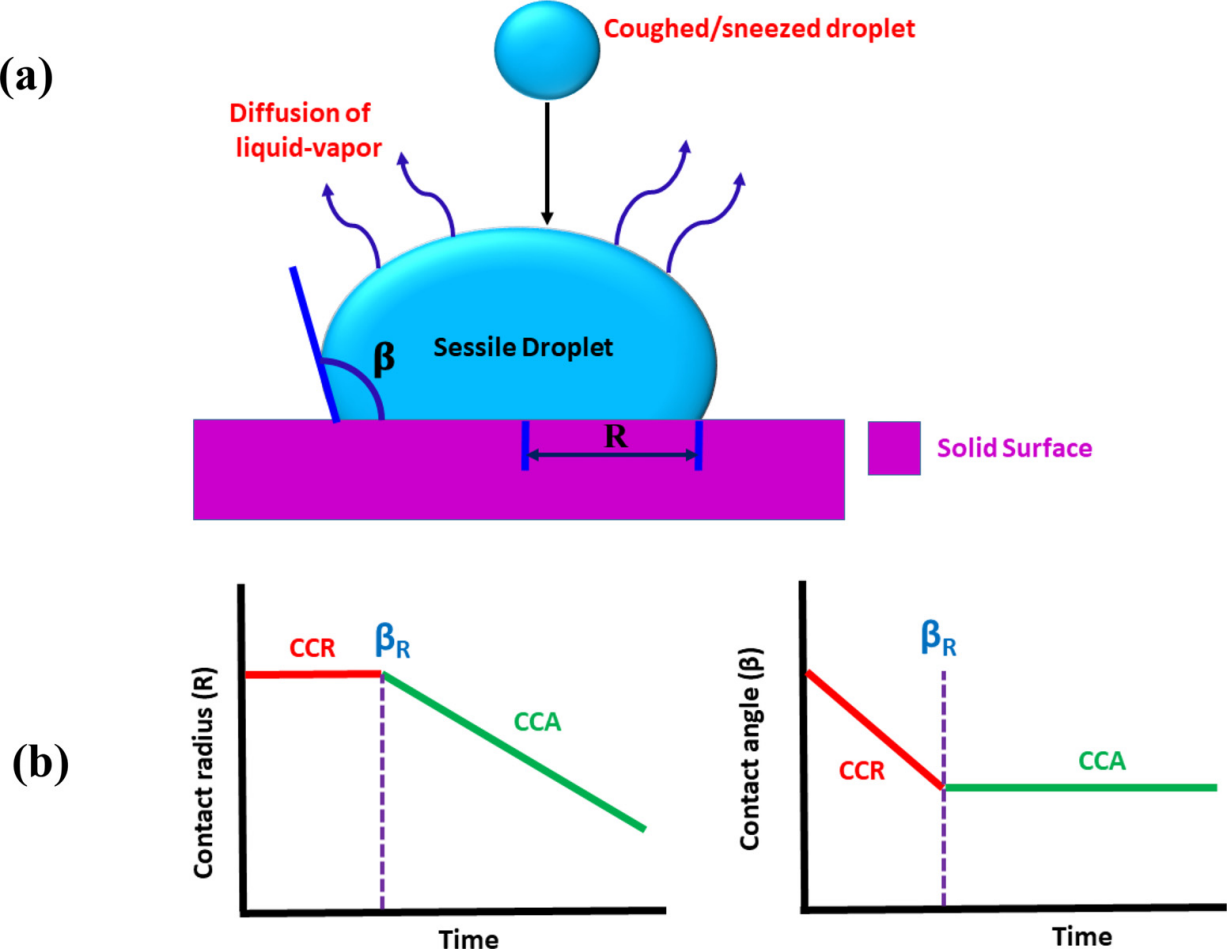
(a) Schematic of the respiratory droplet used to study the survival time of the COVID-19 respiratory droplet under a stick-slip mode and (b) schematic shows the CCR mode and the CCA mode.

Out of all the existing mechanisms for CORONA transmission, respiratory droplets resting on a substrate play a significant role for spreading the infection.^1^ The schematic of such droplets has been shown in Fig. 1. These droplets mostly come out from the mouth when a person sneezes, gets a cough, or even speaks.^1,2^ The total evaporation time of such drops is very crucial, as it decides the duration over which the COVID virus will remain alive and can infect when a person comes in contact with a contaminated droplet.^2,4^ The COVID-19 virus can survive up to a few days on different solid surfaces. Furthermore, the virus needs contact of 5 s in order to get transferred from a surface to a human body.^1,14^ It can thus be assumed that the chance of COVID-19 infection would reduce after the drop gets dried on the surface.

The size of respiratory droplets varies from 20 to 400 *μ*m in diameter.^1–3^ Han *et al.*^15^ investigated the size distribution of drops of 20 healthy subjects during sneezing. The mean diameter reported for sneezed drops was 360 *μ*m. Li *et al.* investigated the flow dynamics during toilet flushing and its influence on the spreading of virus aerosol particles.^16^ They observed that 40%–60% of virus particles reach above the toilet seat that may lead to a large-scale virus spread. In another study, Li *et al.* examined the effects of wind speed, social distancing, and relative humidity on the SARS-CoV-2 viral drop transport during evaporation.^17^ The results reveal that the viral drop of 100 *μ*m can travel up to 6.6 m under a given wind speed of 2 m/s, and this increases further under a dry environment. Wang *et al.* modeling results also corroborate this observation.^12^ Dbouk and Drikakis numerically demonstrated that the human saliva droplets can travel up to 6 m at a 20° ambient temperature and a 50% relative humidity under the speed of the wind changing from 4 to 15 km/h.^7^ In the follow-up work, the researchers' findings emphasize the need for maintaining a social distancing to avoid viral infection as a number of saliva droplets, during sneezing and coughing, gather near the face-mask.^18^ Das *et al.* showed that the smaller droplets can carry the pathogens to a longer distance.^19^ It means maintaining the social distancing of only six feet would not be enough to avoid the COVID-19 viral infection. Busco *et al.* have proposed a computational model in order to understand the human sneezing behavior by considering the realistic model using the combination of the numerical methods and the experiments.^20^ Chatterjee *et al.* have developed a method for designing of antiviral surfaces, which could reduce the survival time of COVID-19 virus.^21^ They have also developed a thin-film model for analyzing the virucidal properties of surfaces. Fontes *et al.* have presented a numerical model to study the droplet dispersion from a sneeze.^22^ During this investigation, they have varied a series of human physiological factors such as illness, anatomy, stress condition, and sex of the individual. This study provides a novel insight and reveals how the transmissibility rate is influenced by the physiological factors. Mirikar *et al.* have numerically investigated the effect of the position of vent, efficacy of the mask, and the ventilation rate on the droplet transmission inside a conference room.^23^ It is observed that the increase in the ventilation rate enhances the droplet extraction rate through the outlet vent.

While most of the existing reports have investigated the role played by coughed or sneezed droplets in spreading the infectious disease, like the COVID-19 infection, the lifetime of COVID-19 droplets residing on the solid surfaces and their dependency on the contact angle^24–26^ have not yet been fully explored. Recently, Bhardwaj and Agrawal have examined the effect of contact angle in reducing the drying time of coronavirus-laden coughed droplets deposited on a solid material.^4^ Their mathematical model unveils that the drying time strongly depends upon the thickness of the thin film. In the follow-up study, the authors investigated the survival time of virus-laden droplets on a partially wetted substrate with a constant contact area mode, and they observed that the survival of the virus primarily affected by the initial contact angle, droplet volume, and ambient temperature.^2^ In another report, the researchers inspected the drying time of COVID-19 droplets on different wettable solid materials, primarily related to the personal protection equipment (PPE) and a face-mask.^1^ They further examined the role of impurities in altering the evaporation time of COVID droplets. However, most of the existing reports on the drying time of COVID-19 droplets have been considered only for the state of a pinned contact line mode (i.e., constant contact area). The effect of a stick-slip mode of evaporation has not considered for getting deep physical insights of the survival time of COVID-19 droplets. As discussed earlier, it would be of crucial significance in order to minimize the chances of COVID-19 infection (which corresponds to the shorter lifetime of the virus-laden droplet) under the stick-slip mode of evaporation (where drying time is affected by both an initial contact angle and a receding contact angle). In actual practice, the droplet drying under the stick-slip mode is highly relevant along with the pinned contact line mode.

Therefore, in the present report, the role of a stick-slip evaporation mode on the lifetime of COVID-19 droplets (i.e., the survival time of COVID-19 virus) has been investigated on dissimilar wettable materials ranging from hydrophilic to hydrophobic. The ratio of the lifetime of a sessile drop on a solid surface to that of a spherical airborne droplet is further examined. This study reveals that the drying time and subsequently the lifetime of the COVID droplet resting on a solid surface decrease with an increasing receding angle. However, the drying time begins to increase beyond a certain threshold value of the receding angle. The proposed models further show that the survival time of respiratory droplets increases with an increasing initial contact angle of the droplet. These interesting observations can help in designing and selecting the materials used for the protection equipment like the PPE kit and the face mask along with the body of different structures used in public places, which can reduce the chances of the COVID-19 infection by increasing the evaporation rate of droplets.

## II. THEORETICAL MODEL

In this paper, a pure diffusion model has been used to develop a mathematical model to determine the lifetime of the sessile respiratory droplet evaporating in the stick-slip (SS) mode. The volume of the respiratory droplets varies from 5 to 10 nl during coughing, sneezing, or speaking.^1–4^ The corresponding diameter of the droplet varies from 214 to 270 *μ*m. In this study, the drop diameter as 214 *μ*m for all the computation has been taken. It is assumed that the droplet preserves a spherical cap shape after coming in contact with a solid surface. As the droplet diameter is much smaller than that of the capillary length, the respiratory droplet remains in a spherical cap during evaporation.^27,28^ Now the volume of a COVID droplet on a solid plate, under these assumptions, can be expressed as

$$V=\frac{\pi }{24}\frac{\left(\;{\text{cos}}^{3}\beta -3\;\text{cos}\;\beta +2\right)}{\;{\text{sin}}^{3}\beta }D^{3},$$ (1)

where *D* and *β* represent the contact diameter and the contact angle of a drying droplet, respectively. Therefore, the evaporation rate of the droplet can be computed as

$$\begin{array}{c} \frac{dV}{dt}=\frac{3\pi D^{2}}{12}\frac{\left(\;{\text{cos}}^{3}\beta -3\;\text{cos}\;\beta +2\right)}{\;{\text{sin}}^{3}\beta }\frac{dD}{dt}+\frac{\pi D^{3}}{8}{\left(1+\text{cos}\;\beta \right)}^{-2}\frac{d\beta }{dt}\\ =\frac{3\pi D^{2}}{4}f(\beta )\frac{dD}{dt}+\frac{\pi D^{3}}{8}{\left(1+\text{cos}\;\beta \right)}^{-2}\frac{d\beta }{dt}, \end{array}$$ (2)

where $f(\beta )=\frac{\;{\text{cos}}^{3}\beta -3\;\text{cos}\;\beta +2}{3\;{\text{sin}}^{3}\beta }$. The lifetime of drying water droplets (>1 s) is much larger than that of its diffusion time (D^2^/D_v_ ∼ $10^{-5}\;\text{to}\;10^{-4}$ s).^29^ Therefore, the evaporation process can be considered as a quasi-steady process.^29^ Moreover, it is also assumed that the thermal and convection effects remain negligible, and therefore, it can be considered that the evaporation of the COVID-19 virus drop is purely diffusion-driven. Under the above discussed assumptions, the evaporation rate of a droplet can be written as follows:^30,31^

$$\frac{dV}{dt}=-\pi D\frac{D_{V}C_{S}(1-H)}{\rho }{\left(1+\text{cos}\;\beta \right)}^{-\frac{1}{2}}=-\frac{\pi D\lambda }{\rho }g(\beta ),$$ (3)

where $g(\beta )={\left(1+\text{cos}\;\beta \right)}^{-\frac{1}{2}}$ and $\lambda =D_{V}C_{S}(1-RH)$. $D,\;D_{V},C_{S},\;\mathrm{and}\;\;H$ are the droplet wetted diameter, diffusion coefficient of water-vapor in air (m^2^/s), saturated water vapor concentration (kg/m^3^), and relative humidity, respectively. In terms of the mass loss, $\dot{M}$, Eq. (3) can be further expressed as

$$\dot{M}==-\pi D\lambda {\left(1+\text{cos}\;\beta \right)}^{-\frac{1}{2}}.$$ (4)

The concentration of saturated water-vapor (kg/m^3^) at a given temperature (*T*, °C) has been determined using the polynomial fitting of available data in the existing report,^1^

$$C_{S}=4.35\times 10^{-9}T^{4}-4.53\times 10^{-8}T^{3}+1.79\times 10^{-5}T^{2}+2.35\times 10^{-4}T+5.07\times 10^{-3}.$$ (5)

The diffusion coefficient of water-vapor ( $D_{V}$, m^2^/s) as a function of an ambient temperature (*T*, °C) can be determined by the following expression:^1^

$$D_{V}(T)=2.5\times 10^{-4}\;\text{exp}\;\left(-\frac{684.15}{T+273.15}\right).$$ (6)

Before developing the equation for the total evaporation time of a COVID drop in the stick-slip (SS) mode, it is essential to first determine the expression for a constant contact diameter mode (CCD) (also known as a constant contact area or a constant contact radius mode) and a constant contact angle (CCA) mode. The relationship between the contact angle and the drying time in the CCD mode can be determined using Eqs. (2) and (3) along with a simple mathematical computation as

$$t(\beta )=\frac{\sqrt{2}}{32}\frac{\rho D_{0}{}^{2}}{\lambda }[P(\beta_{0})-P(\beta )],$$ (7)

where β_0_ represents the initial contact angle of the droplet. The function P(β) is given by

$$P(\beta )=\text{ln}\;\left[\text{tan}\;\left(\frac{\pi }{4}+\frac{\beta }{4}\right)\right]+\text{sin}\;\frac{\beta }{2}{\left(\;{\text{cos}}^{2}\frac{\beta }{2}\right)}^{-1}.$$ (8)

Now the total evaporation time or the lifetime of the COVID droplet in the pinned contact line mode (CCD mode) can be obtained using Eq. (7) when β = 0 as

$$T_{\mathrm{CCD}}=\frac{\sqrt{2}}{32}\frac{\rho D_{0}{}^{2}}{\lambda }P(\beta_{0}).$$ (9)

In the similar way, the relationship of a contact diameter with time under the CCA mode can be derived using Eqs. (2) and (3) and considering D = 0 can be expressed as^28^

$$t(D)=\frac{3}{16}\frac{\rho }{\lambda }\frac{f(\beta_{0})}{g(\beta_{0})}(D_{0}{}^{2}-D^{2}).$$ (10)

The lifetime of the droplet evaporating under the CCA mode [using Eq. (10) and considering D = 0] can be obtained as^28^

$$T_{\mathrm{CCA}}=\frac{3}{16}\frac{\rho }{\lambda }\frac{f(\beta_{0})}{g(\beta_{0})}D_{0}{}^{2}.$$ (11)

The lifetime, T_SS_ of a virus-laden droplet drying in a stick-slip mode depends upon the three parameters, namely, the initial contact angle (*β_0_*), contact diameter (*D*_0_), and receding angle (*β_R_*). The equation for determining the lifetime of a sessile evaporating drop under the SS mode can be derived using Eqs. (9) and (11) and can be expressed as^28^

$$T_{SS}=\frac{\sqrt{2}}{32}\frac{\rho D_{0}{}^{2}}{\lambda }[P(\beta_{0})-P(\beta_{R})]+\frac{3}{16}\frac{\rho }{\lambda }\frac{f(\beta_{R})}{g(\beta_{R})}D_{0}{}^{2},\;0\leq \beta_{R}\leq \beta_{0}.$$ (12)

Equation (12) can be further expressed in terms of the drying time of the CCD mode, $T_{\mathrm{CCD}}$ and the CCA mode, $T_{\mathrm{CCA}}$ as given by^28^

$$T_{SS}=T_{\mathrm{CCD}}\left(1-\frac{G(\beta_{R})}{G(\beta_{0})}\right)+T_{\mathrm{CCA}}\frac{g(\beta_{0})}{f(\beta_{0})}\frac{f(\beta_{R})}{g(\beta_{R})},\;0\leq \beta_{R}\leq \beta_{0}.$$ (13)

In this investigation, the lifetime of a sessile droplet evaporating under the stick-slip mode is further compared with that of an airborne drop by keeping the volume same. The lifetime of an airborne droplet can be derived using the rate of mass loss of a spherical drop and can be written as^1,32,33^

$$T_{ab}=\frac{\rho D_{ab}{}^{2}}{8\lambda },$$ (14)

where $D_{ab}$ is the diameter of the airborne droplet, which is equal to the initial diameter of a sessile droplet (*D_0_*). Now, the ratio of the lifetime of two drops, $\frac{T_{SS}}{T_{ab}}$ using Eqs. (13) and (14) can be stated as follows:

$$\frac{T_{SS}}{T_{ab}}=\frac{\sqrt{2}}{4}[P(\beta_{0})-P(\beta_{R})]+\frac{3}{2}\frac{f(\beta_{R})}{g(\beta_{R})},\;0\leq \beta_{R}\leq \beta_{0}.$$ (15)

The above equation reveals that the ratio of the lifetime of an evaporating sessile drop under the stick-slip mode with that of the airborne droplet is only a function of the initial contact angle and the receding angle. It means that the ambient temperature, the relative humidity, the thermos-physical properties, and the initial volume of the drop do not play any role in altering this ratio.

## III. RESULTS AND DISCUSSION

Here, the results obtained using the proposed analytical model have been presented for the receding angle and the initial contact angle varying in the range of 1° < β_R _< 150° and 5° < β_0 _< 100°, respectively. The properties of the water droplet for the computation of the lifetime of the drying droplets under the stick-slip mode have been used. As the thermo-physical properties of the COVID-19 saliva droplet and the water droplet are almost the same, the reported findings offer a good physical understanding and the total evaporation time of the coughed drops on the different types of solid surfaces.

### A. Validation of the model

In order to verify the mathematical model, the lifetime of a drop [using Eq. (13)] evaporating on a hydrophilic surface in the SS mode has been compared with the experimental data reported in Nguyen *et al.*^34^ The parameters used for the validation purpose are as follows: β_0 _= 58°, β_R _= 32°, D_0 _= 3.0 mm, H = 0.55, T = 25 °C, and D_V_= 2.52 × 10^−5^ m^2^/s. It is observed that the result of the model (T ∼ 1700 s) shows a good agreement with the experimental observation (T ∼ 1600 s) of Nguyen *et al.*^34^ It is further computed and compared with the lifetime of a drying droplet (D = 3.4 mm) on a glass surface with β_0 _= 29°, T = 27 °C, and H = 35%. In this report, the droplet was remained pinned for 90 of the total lifetime of the droplet. The experimentally measured drying time of the drop is 632 s,^35^ while the computationally predicted lifetime for this case is 601[using Eq. (13)]. A good prediction of the experimental findings^35^ by the proposed model further confirms the robustness of the model.

### B. The influence of the SS mode on the lifetime of COVID-19 droplets on different wettable substrates

**FIG. 2.**
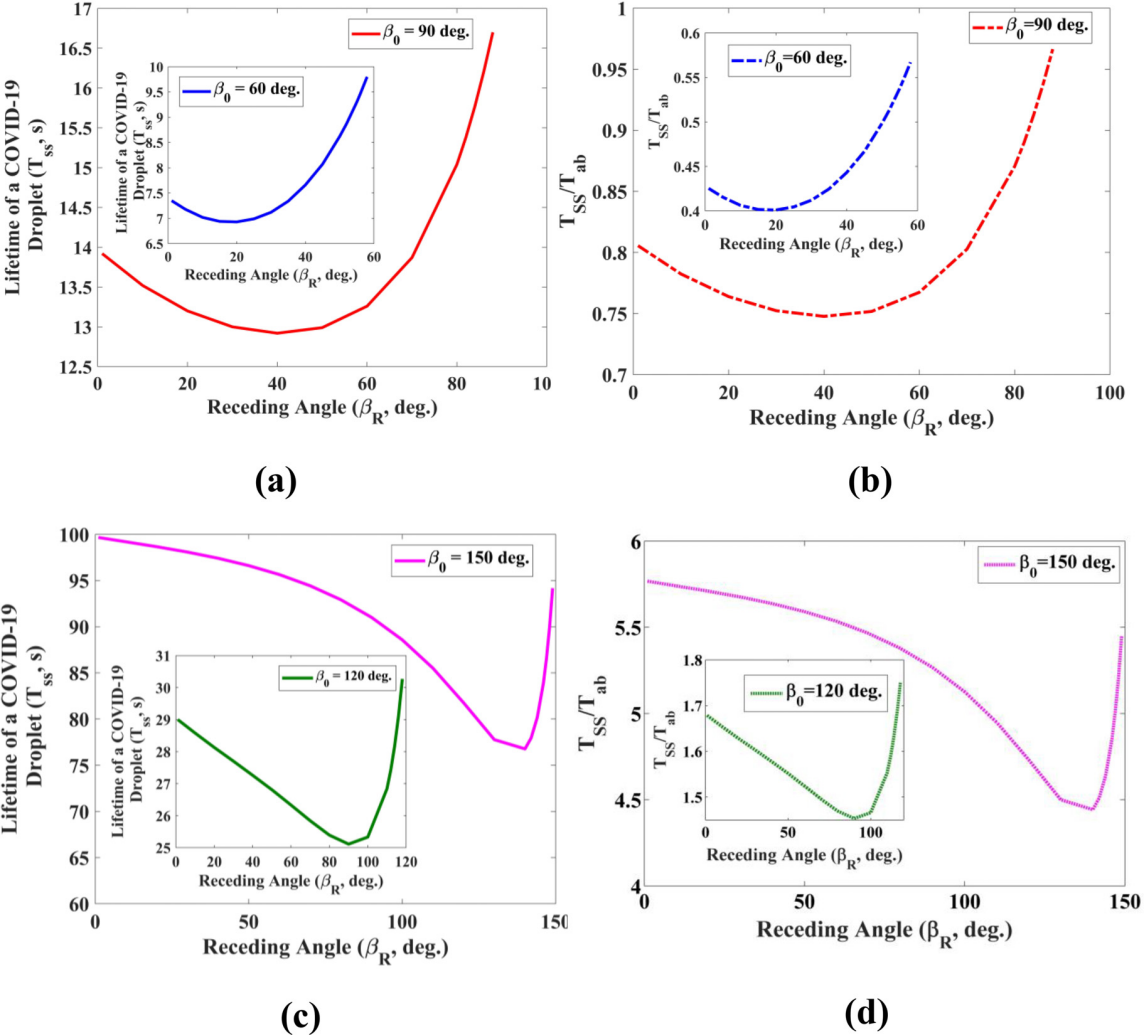
Lifetime (survival time) of evaporating droplet on a solid surface as a function of the receding angle (**β*_R_*) and with varying wettability {[(a) and (b)] 60° and 90°, [(c) and (d)] 120° and 150°}. The volume of the droplet taken here is 5 nl (radius of a drop, R = 214 *μ*m). The relative humidity, H and the ambient temperature, T taken for the computation are 50% and 27 °C, respectively.

**FIG. 3.**
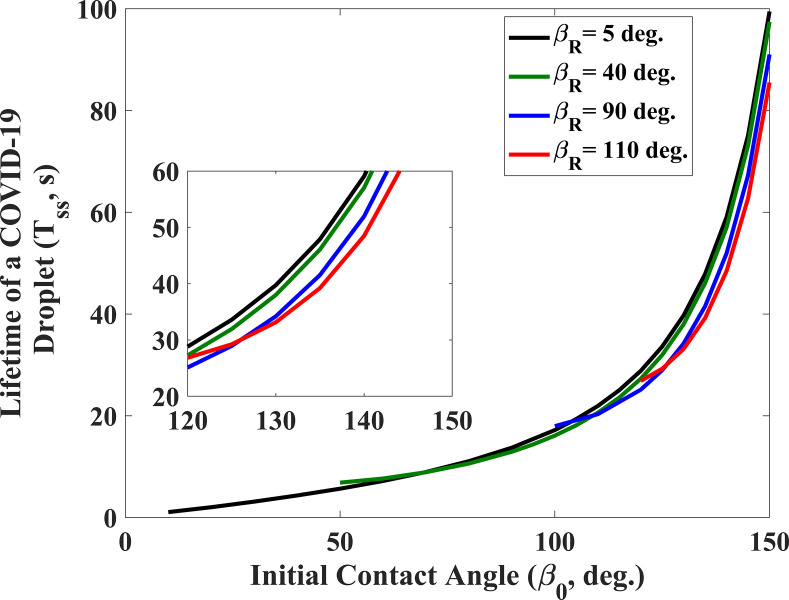
Lifetime (survival time) of evaporating droplet on a solid surface as a function of the initial contact angle (**β*_0_*) of the drop for different receding angles (**β*_R_*). The volume of a droplet taken here is 5 nl (radius of a drop, R = 214 *μ*m). The relative humidity, H and the ambient temperature, T taken for the computation are 50% and 27 °C, respectively.

Figure 2 depicts the lifetime of the COVID-19 droplet with the receding contact angle under the stick-slip mode. The lifetime of the respiratory droplet decreases with increasing receding contact angle. However, the drying time begins to increase beyond a certain threshold value of the receding contact angle. In fact, the increase in the drying time happens when the initial contact angle and receding angle are very close to each other. It implies that the droplet gets evaporated primarily in the CCA mode or the mixed mode in most of the time. As a result, the lifetime of the drying droplet increases as the receding contact angle reaches closer to the initial contact angle of the droplet. In this investigation, four types of wettable substrates (β_0_ = 60°, 90°, 120°, and 150°) have been considered in order to determine the lifetime of coughed or sneezed drops. The plot in Fig. 3 further reveals that the lifetime of a drop increases with an increasing initial contact angle of the droplet. In another way, the drying rate of droplets decreases as the substrate becomes hydrophilic to superhydrophobic. This observation suggests that the substrate can be made more hydrophilic in order to augment the evaporation rate of respiratory droplets. However, surfaces of different structures used in various public places do not always possess a hydrophilic nature. It means that it would not always be possible to make all surfaces hydrophilic to reduce the drying time due to practical constraints. In this scenario, the material can be re-designed by choosing a suitable value of the receding angle and the wettability (using Fig. 2) for obtaining a small drying time of the respiratory droplet. Moreover, the lifetime and chances of infection can be reduced by increasing the surface roughness of the hydrophilic surface as it further augments the hydrophilic nature of the substrate if it is in the Wenzel state.

**FIG. 4.**
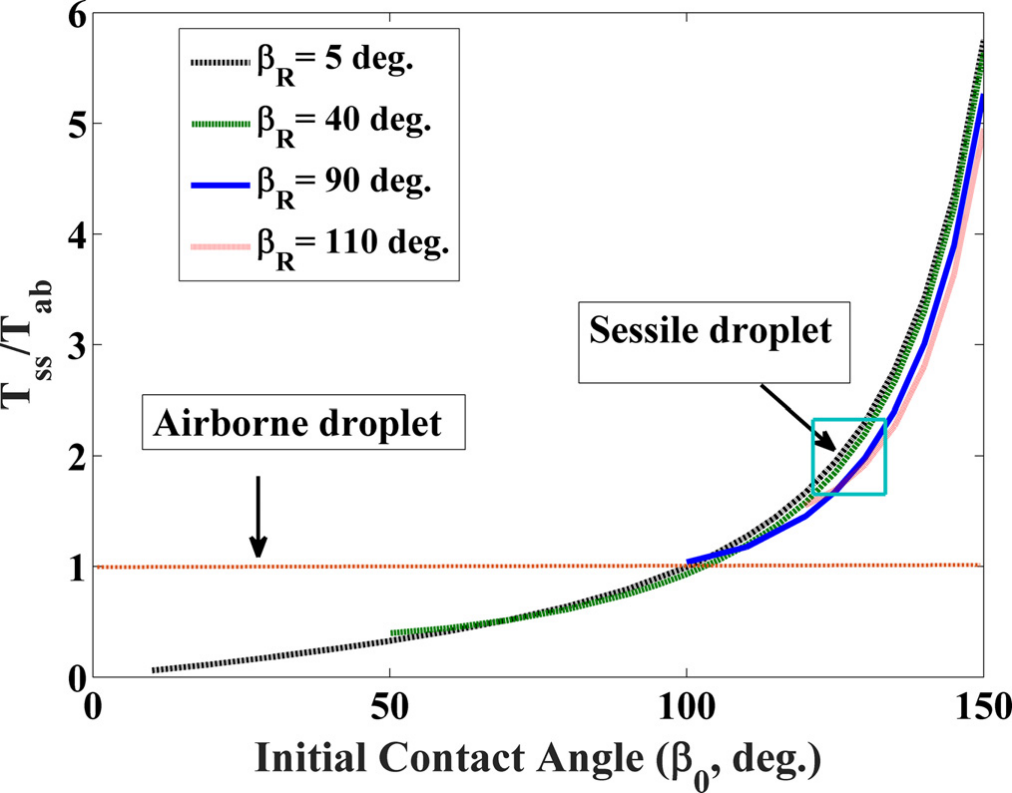
Normalized lifetime (survival time) of a droplet on a solid surface to that of a spherical airborne droplet.

Figure 4 illustrates the ratio of the lifetime (T_SS_) of a respiratory droplet on a solid surface to that of the airborne droplet (T_ab_) of the same volume. This plot unveils that the lifetime of a sessile droplet is higher than that of the airborne droplet except for the cases when the contact angle, *β_0_*_ _< 100°. This may be attributed to the fact that the mass of the vapor does not diffuse from one side of the droplet due to the presence of a solid substrate, which leads to an enhancement up to 400% in the lifetime of a drying droplet for 100° < *β_0_*_ _≤ 150°. However, an opposite trend is observed for the surfaces, where the contact angle is less than 100°. In this type of a droplet-solid system, the wetted diameter has been found to be large, leading to subsequent augmentation in the surface area of the drop. The increased surface area of a drop compensates for the loss that occurred due to the presence of a solid surface, which leads to a reduction in the lifetime.

It is important to mention that the universal lifetime curve shown in Fig. 4 is able to predict the lifetime of all ranges of respiratory droplets produced through sneezing, coughing, or speaking. The diameter of the respiratory droplet, in general, varied from 20 to 800 *μ*m. The present theoretical model suggests that the total evaporation time of coughing droplets on a solid surface (with *β_0_* = 150°, *β_R_* =50° and H = 50%] varies from 3.37 to 1350 s.

**FIG. 5.**
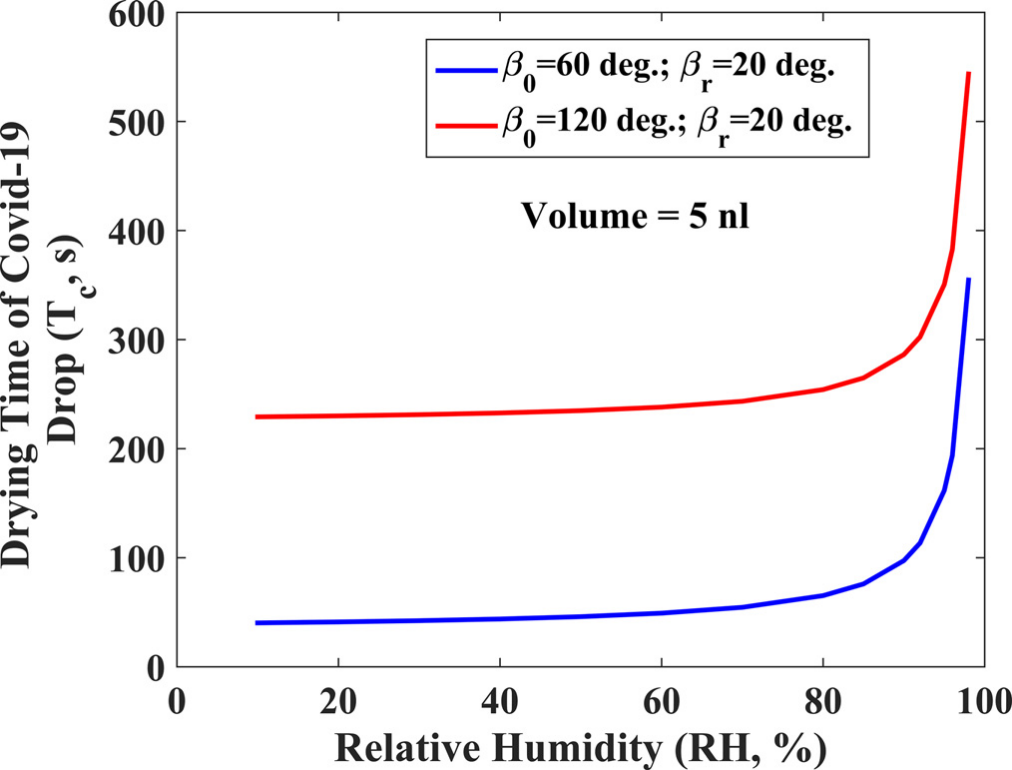
Variation of the drying time of the COVID-19 droplet with relative humidity.

The survival time (or lifetime) of airborne coronavirus has been found to be larger than several other viruses at a lower relative humidity of 20%–30%, as the SARS-CoV-2 virus is enclosed with a protective lipid layer. In Fig. 5, the role of relative humidity has been studied on the survival time of virus present inside a respiratory droplet deposited on a solid surface for the two cases of wettability and the receding contact line. The plot in Fig. 5 reveals that the relative humidity plays an important role in controlling the survival time of virus-laden COVID droplets. The drying time of the droplet increases with an increase in the relative humidity (RH, %). The lifetime of droplets increases almost five times when relative humidity increases from 10% to 90% at a solid surface with contact angle, *β_0_*_ _= 60°, *β_R_*_ _= 20°, and T = 27 °C. In a similar way, the increase in drying time is approximately 1.5 times for the substrate, where *β_0_*_ _= 120°, *β_R_*_ _= 20°, and T = 27 °C.

### C. The influence of diffusion-driven flow and thermal Marangoni flow on the shear stress acting on the coronavirus in a respiratory droplet deposited on a solid surface

**FIG. 6.**
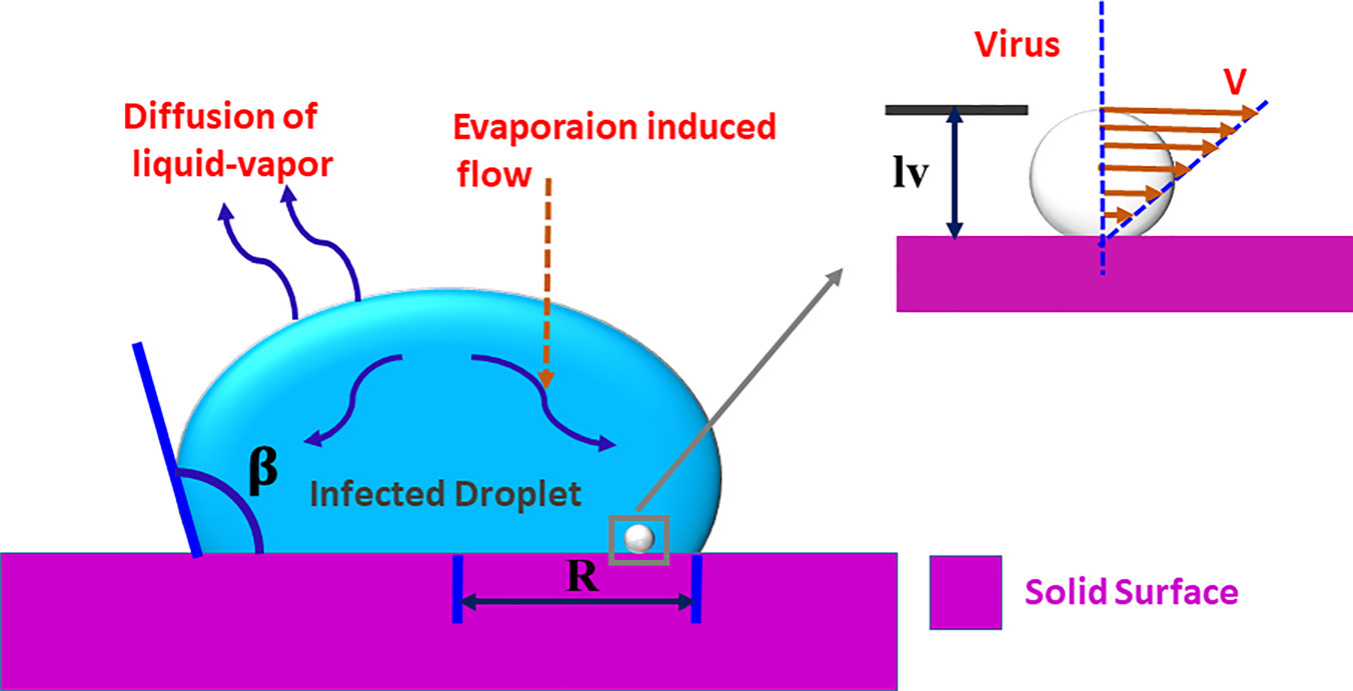
Schematic of the virus-laden droplet. In this study, we estimate the shear stress acting on a virus due to the evaporation induced flow and the Marangoni induced flow.

In this section, the maximum shear stress acting on the SARS-CoV-2, suspended in the sessile water droplet has been examined. As the thermo-physical properties of saliva and water are almost the same, the properties of water have been considered for determining the shear stress. The knowledge of the order of magnitude of shear stress acting on a virus is crucial as it will give a rough idea about the survival time of the COVID virus. The maximum stress on a virus would act when it would have adhered to the solid surface (Fig. 6). Now considering a linear velocity profile across the virus cross section, the shear stress, *τ* can be expressed as^2^

$$\tau =\eta \frac{V}{l_{v}},$$ (16)

where η, V, and $l_{v}$ are the viscosity of drop, flow velocity on the apex of a virus, and diameter of the virus, respectively (Fig. 6). The expression for evaporative mass flux on the liquid–air interface by taking into account the effect of thermal Marangoni stress, J, (kg/m^2^s), can be written as^36^

$$J(r)=-\frac{D_{V}C_{S}(1-R_{H})}{R}(0.27\beta_{0}{}^{2}+1.30)\times \left(-1.407\times 10^{-4}\frac{\partial \gamma }{\partial T}\frac{L\Delta T}{\eta \alpha }+1\right).$$ (17)

In order to compute the value of the term, $\frac{\partial \gamma }{\partial T}$, the relationship of surface tension with temperature should be known. The surface tension, *γ* in terms of temperature, T can be given as^37^

γ=−0.000 164T+0.0759. (18)

The maximum evaporating mass flux (J_m_) occurs near the contact line of the drop.^2^ Therefore, the evaporation-driven mass flow velocity, V can be expressed as follows:

$$V=\frac{J_{m}}{\rho }.$$ (19)

Now the maximum shear stress acting on the virus adhered to the solid surface has been therefore derived using Eqs. (16)–(18) as

$$\tau =\frac{\eta V}{l_{v}}=\frac{\eta J_{m}}{\rho l_{v}}.$$ (20)

**FIG. 7.**
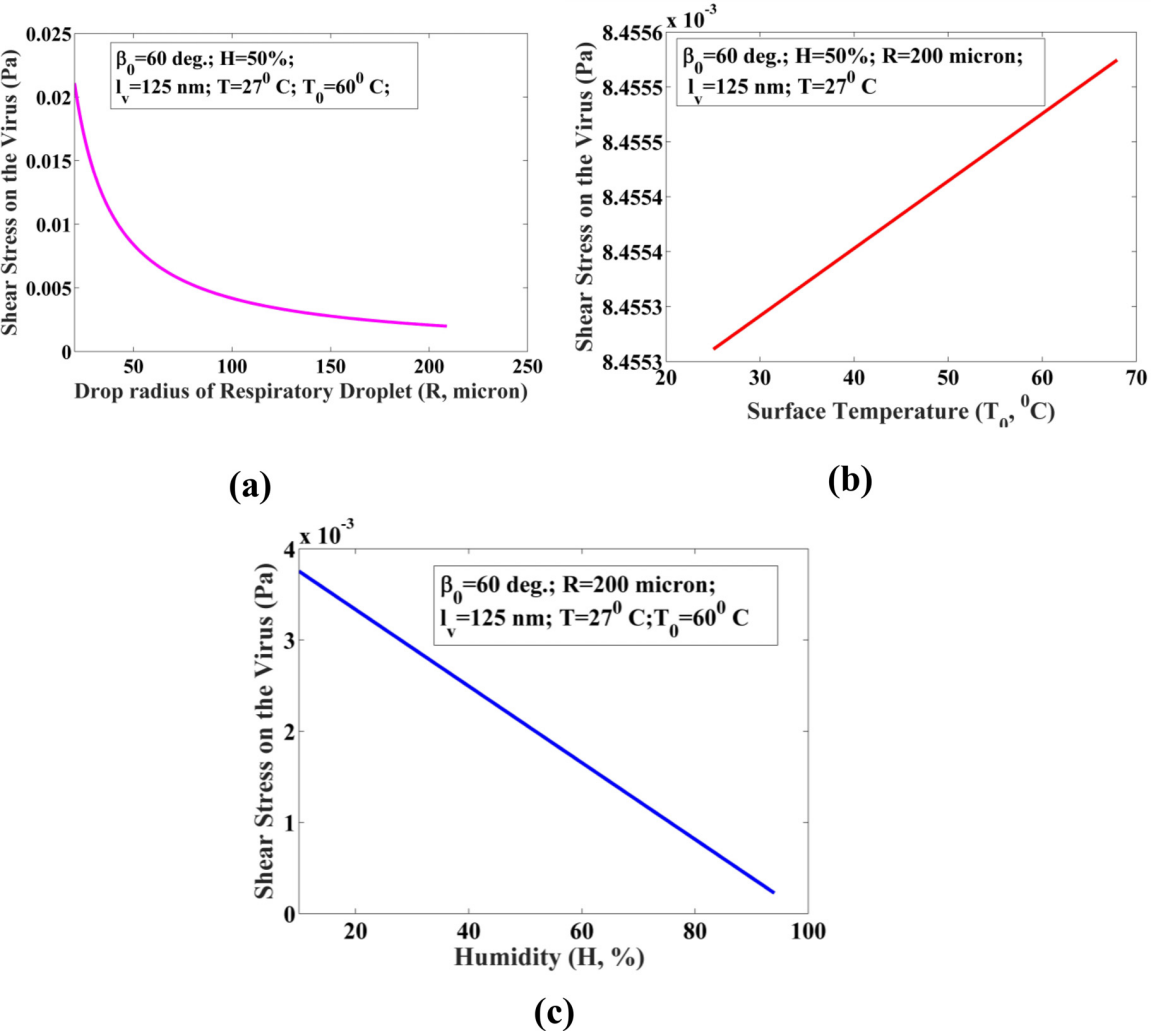
The variation of the shear stress on a virus suspended inside a droplet deposited on the solid surface. The shear stress acting on a virus has been plotted with (a) drop radius, R; (b) solid surface temperature, T; and (c) humidity, H of the ambient. All other parameters used for computation have been given in the plot.

The magnitude of the shear stress acting on the virus suspended in the droplets of volume 33 pl to 34 nl has been estimated at β_0 _= 60°, H = 50%, T = 27 °C, and T_0 _= 60 °C using Eq. (20) (Fig. 7). The corresponding diameter varies from 20 to 200 *μ*m. The plot in Fig. 7(a) unveils that the shear stress acting on a virus decreases with the increase in the size of the respiratory droplet. However, the shear stress increases with an increasing temperature of the hydrophilic surface. The reason behind this augmentation in the shear stress is the thermal Marangoni stress, which occurred due to the increasing temperature gradient across the surface of coughed droplet [Fig. 7(b)]. Moreover, the humidity of the ambient plays a crucial role in modulating the shear stress on the virus. Figure 7(c) suggests that shear stress can be increased by reducing the humidity of the surroundings. It is highly desirable to keep the shear stress acting on a virus higher as it may kill the virus if the stress is sufficiently high.

The COVID-19 virus present inside the saliva droplet encounters shear stresses due to the evaporation-induced flow, which may be due to all three cases discussed above. The flow-induced inside a droplet may purely be diffusion-driven or the combination of the Marangoni flow and the diffusion-driven flow. Based on the observation in Fig. 7, it can be established that the magnitude of shear stress varies from 0.000 25 to 0.02 Pa for the considered cases. However, the magnitude of the maximum shear stress acting on a virus is not sufficient to disrupt the virus inside the droplet. Therefore, in order to reduce the COVID-19 infection, the minimization of drying time of virus-laden droplets is very significant as the shear stress alone would not be able to kill the virus.

Finally, we discuss the importance of this study from the perspective of reducing the COVID-19 infection. The lifetime of the respiratory droplet is one of the most significant parameters, which tells the duration over which a person can get infected if he or she comes in contact with a virus-infected droplet. The virus cannot live without a liquid medium, and therefore, it is considered that there would be a very less chance of the COVID-19 disease if the virus-laden droplet gets evaporated. This is the reason that the lifetime droplet also represents the survival time of a virus. The findings reveal that the survival time of the COVID-19 virus primarily depends upon the wettability of the surface on which the virus-laden droplet falls, mode of evaporation, humidity, and temperature of the surroundings. The physical insight of the survival time of droplets may help in the re-design of the face mask and the PPE kit. The results reported in this paper can be used to reduce the survival time of the COVID-19 droplet deposited on the surface of the face mask or the PPE kit. The materials and design of the protection kit can be chosen by optimally tailoring the wettability of the surface and the receding contact angle.

### D. Limitations of the model

The presented model in this paper is able to predict the survival time of COVID-19 droplets with good accuracy. However, this model has a few limitations. The thermo-capillary convection effect (the Marangoni effect) in the model has been neglected, which predicts the lifetime of an infected droplet in the stick-slip mode. The effect of the thermo-capillary convection on the lifetime of COVID-19 droplet is beyond the scope of this paper and will be investigated in the future as separate work. The presence of any solute in the saliva droplet and its effect on the survival time has not been taken into consideration. However, it is expected that the survival time of the droplet will not get altered much due to the presence of the solute. Moreover, the effect of solute present in the saliva droplet on the lifetime may be investigated as an extension of this work. We have considered the effect of thermo-capillary convection (the thermal Marangoni flow) for the model proposed to estimate the magnitude of shear stress acting on a virus.

## IV. CONCLUSION

In summary, a mathematical model has been developed to determine the survival time or lifetime of virus-infected droplets under the stick-slip mode, which may play a critical role in reducing the spreading of COVID-19 infection. In order to get physical insight on the lifetime of the drying droplet, wettability of the substrate and the receding contact angle has been varied over a wide range. It is revealed that the survival time of the virus can be reduced either by increasing the receding contact angle or by reducing the initial contact angle of the drop deposited on a solid surface. The survival time of the virus increases almost five times under highly humid conditions as compared to dry conditions. It is further found that the normalized lifetime is independent of the thermo-physical properties, droplet initial volume, ambient temperature, and relative humidity. In addition, a model to estimate the shear stress acting on a virus has been proposed. The presented model unveils that the magnitude of computed shear stress is not enough to kill the virus. In this paper, although the findings of the theoretical model have been discussed in the context of reducing the COVID-19 infection, the model can also be applied for coughed/sneezed droplets of other infectious diseases. Moreover, this physical understanding of evaporation dynamics on solid surfaces with the stick-slip mode may help in better design of the face mask, PPE kit, and other protection equipment in order to minimize the chances of infection and tackle the current pandemic.

However, the reported model for estimating the survival time of the virus does not take into account the effect of the thermo-capillary convection (the Marangoni effect) effect. The presence of any solute in the saliva droplet and its effect on the survival time has not been taken into consideration. However, it is expected that the survival time of the droplet will not get altered much due to the presence of the solute.

## Data Availability

The data that support the findings of this study are available from the corresponding author upon reasonable request.
